# Basics and recent advances of two dimensional- polyacrylamide gel electrophoresis

**DOI:** 10.1186/1559-0275-11-16

**Published:** 2014-04-15

**Authors:** Sameh Magdeldin, Shymaa Enany, Yutaka Yoshida, Bo Xu, Ying Zhang, Zam Zureena, Ilambarthi Lokamani, Eishin Yaoita, Tadashi Yamamoto

**Affiliations:** 1 Department of Structural Pathology, Institute of Nephrology, Graduate School of Medical and Dental Sciences, Niigata University, 1-757 Asahimachi-dori, Niigata, Japan; 2 Department of Physiology, Faculty of Veterinary Medicine, Suez Canal University, Ismailia, Egypt; 3 Department of Microbiology and Immunology, Faculty of Pharmacy, Suez Canal University, Ismailia, Egypt; 4 UKM Medical Centre, Kuala Lumpur, Malaysia

**Keywords:** Two dimensional electrophoresis, Advances, Basics, Review

## Abstract

Gel- based proteomics is one of the most versatile methods for fractionating protein complexes. Among these methods, two dimensional- polyacrylamide gel electrophoresis (2-DE) represents a mainstay orthogonal approach, which is popularly used to simultaneously fractionate, identify, and quantify proteins when coupled with mass spectrometric identification or other immunological tests. Although 2-DE was first introduced more than three decades ago, several challenges and limitations to its utility still exist. This review discusses the principles of 2-DE as well as both recent methodological advances and new applications.

## Introduction

Two dimensional polyacrylamide gel electrophoresis (2-DE) is considered a powerful tool used for separation and fractionation of complex protein mixtures from tissues, cells, or other biological samples. It allows separation of hundreds to thousands of proteins in one gel. This technique became more popular and comprehensive after the prime advances and high resolution 2-DE modification [1]. Actually, the modification established by O’Farrell allowed 2-DE technique to resolve up to 5000 protein- representing spots in an even 2 dimensional distribution and enabled precise separation of protein spots with high accuracy [2].

2-DE technique is deemed to be one of the leading powers in the expansion of proteomics and protein studies. It provides the first step for further analysis of the differentially regulated protein spots using mass spectrometry and western blotting. The use of 2-DE has been effectively defined in many cases to disclose both physiological mechanisms and proteins associated with clinical pathologies that can aid in the discovery of biomarkers.

## Basic principles of 2-DE

### Concept of 2-DE

2-DE consists mainly of two steps of separation; first dimension and second dimension. In the first dimension, protein molecules are resolved depending on their isoelectric point (pI) [2]. Separation of proteins under a pH gradient allows intense band recovering using various tactics such as immobilized gradient electrophoresis (IPEG), isoelectric focusing (IEF), or non-equilibrium pH gradient electrophoresis (NEPHGE). In the second dimension, protein separation is performed based on molecular weight using SDS Laemmli or Tris-Tricine buffers. Due to the fact that it is improbable that different protein molecules may have the same physicochemical properties (pI and MW), proteins are efficiently separated by 2-DE rather than 1D-SDS PAGE [2]. A prominent merit of 2-DE is that the resolution acquired during the first dimensional separation is not missed in the second electrophoresis when IEF gel strip is connected to the SDS- PAGE gel [2,3].

### Applications and utilities of 2-DE

2-DE is a powerful and widely used method for analysis of complex protein mixtures with exceptional ability to separate thousands of proteins at once. It provides direct visual confirmation of changes in protein/post-translational modifications (PTMs) abundance, thus providing early justification for downstream analytical steps through detecting post- and co-translational modifications, which cannot be predicted from the genomic sequence. Other applications of 2-DE include whole proteome analysis [4], cell differentiation [5], detection of biomarkers and disease markers, drug discovery, cancer research [6], bacterial pathogenesis [7], purity checks, microscale protein purification, and product characterization.

### Advantages and strengths of 2-DE

#### Robustness

During the last few years, several methodological improvements have contributed to increase the robustness of 2-DE workflows. The use of immobilized isoelectric focusing (IEF) strips, ampholytes- based buffers, highly sensitive dyes, and gel imaging software made the variability most likely from upstream process such as protein loss during extraction [8]. In a recent multi-laboratory study on the feasibility of 2-DE, it is reported that 70-93% of spots were detected with coefficient of variation (CVs) less than 20% within same laboratory researchers [9]. On the other hand, 72% of spots showed CVs with less than 20% across laboratories [9]. This finding proves the feasibility and the robustness of 2-DE. Moreover, 2-DE becomes less variable when multiplexing electrophoresis developed. Differentially labeled samples run at the same time minimized the possibility of artifacts resulted from technical errors. Finally, the recent improvements of the gel image analysis minimized the former high percentage of spot identification failure estimated to reach 60% which considered one of the major contributors to variability seen with 2-DE [10].

#### Visualized mapping analysis

**Figure 1:**
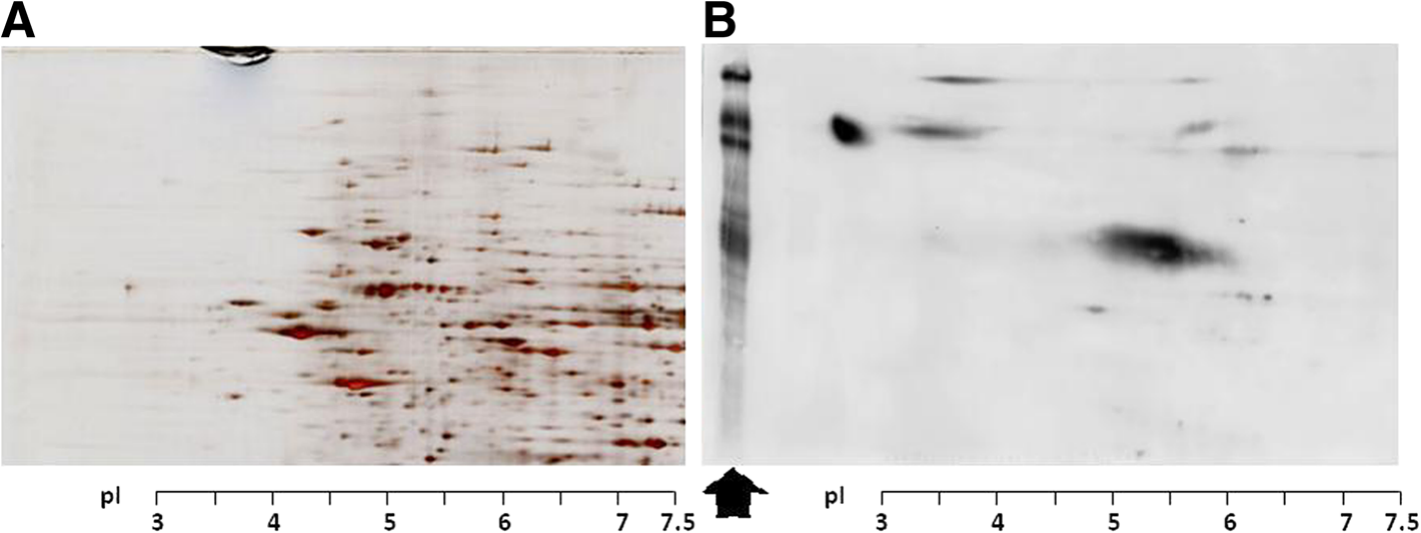
**2-DE showed the resolving of glomerular proteins using 7 cm IPG strip. (A)** Gel stained with silver stain **(B)** western blot of PVDF membrane using P- Tyr- 100 antibody for detecting phosphorylated proteins. Arrow shows 1D- SDS pattern [32].

One of the unique features of 2-DE is its ability to resolve intact full-length proteins (up to 5000 protein) in a single gel. This includes visualized detection of the physico-chemical properties such as MW and pI with possible quantification based on the spot intensity [8]. Proteins of interest could be characterized via peptide mass finger printing or when probed with antibodies. Moreover, 2-DE allows separation and identification of PTMs and protein isoforms (Figure 1). In several cases, PTMs could be recognized by horizontal or vertical shifting of a protein spot as these modifications usually change the protein MW and pI [11].

#### Compatible platform for further analysis

2-DE gel easily and efficiently couples with many other analysis and biochemical techniques. Thus, it provides a compatible platform for subsequent analysis. For example, stained gels can be followed by spot excision, destaining, protein extraction, digestion, and analysis of the tryptic peptides by mass spectrometry. Although coomassie blue could be reversibly destained and compatible with mass spectrometry, silver staining, it is not compatible because of the usage of formaldehyde or glutaraldehyde during the fixing and sensitization step that results in lysine residue cross-linking within the protein chain interfering MS analysis and thus will hinder trypsin digestion [12,13]. Various modifications in the silver nitrate stain approach were performed to overcome this drawback. Compatibility of 2-DE includes bottom-up proteomics to identify proteins and characterize their amino acid sequences or alternatively proceeded by the top-down proteomics (shotgun) in which the crude proteins extract is digested directly for analysis. In another powerful combination, antibody- based analysis could be coupled before or after 2-DE. For instance, immune-affinity purification can be used to pre-fractionate a protein of interest prior to running 2-DE such as phosphorylated [2] or ribonucleoproteins [14]. Most commonly, 2-DE fractionated proteins are subjected to either in-gel digestion to prepare tryptic peptides for mass spectrometric analysis or gels are validated for protein of interest using western blotting [15].

### Limitations of 2-DE

#### Low reproducibility

In the traditional method of the first dimension IEF, the carrier ampholytes is utilized to build pH gradient. The carrier ampholytes-based pH gradient made from soft unsupported tube gels (typically 4% acrylamide) is not stable. There is batch to batch variability and prone to cathodic drift (a progressive loss of basic proteins during long running of electro-focusing under electric field), leading to low reproducibility and requiring careful monitoring of electric field [2]. Replacement of carrier ampholyte-based pH gradient in tube gel with the immobilized pH gradients (IPG) was the key development in increasing the reproducibility of 2-DE [16,17].

#### Difficulty in separating hydrophobic and extremely acidic or basic proteins

Different types of proteins can always be missing, due to the difficulty in separating membrane- bound (hydrophobic) and extreme proteins [18,19]. Notably, highly acidic or basic proteins are neither easily extracted nor solubilized. This difficulty in extraction relies mainly on the solubilization power of the buffer used in the IEF step. Many efforts have been performed for better solubilization of membrane proteins using different chaotropes or detergents. For instant, Triton X- 114 and CHAPS showed a powerful recovering power towards hydrophobic proteins on 2-DE when tested immunologically [20]. Another study reported the use of cationic detergent benzyldimethyl-n-hexadecylammonium chloride to improve resolving hydrophobic proteins with GRAVY index as low as 0.56 [21]. Similar improvement was reported when using detergents such as DHPC [22] and 1,4-dithioethanol [23]. Although solubilization of wide range of proteins could be achieved using denaturating solution (Urea and Thiourea) and zwitterionic detergents (SB 3–10) [24], resistance of certain proteins still remains a built-in problem [2,24].

#### Narrow dynamic range of 2-DE

**Figure 2:**
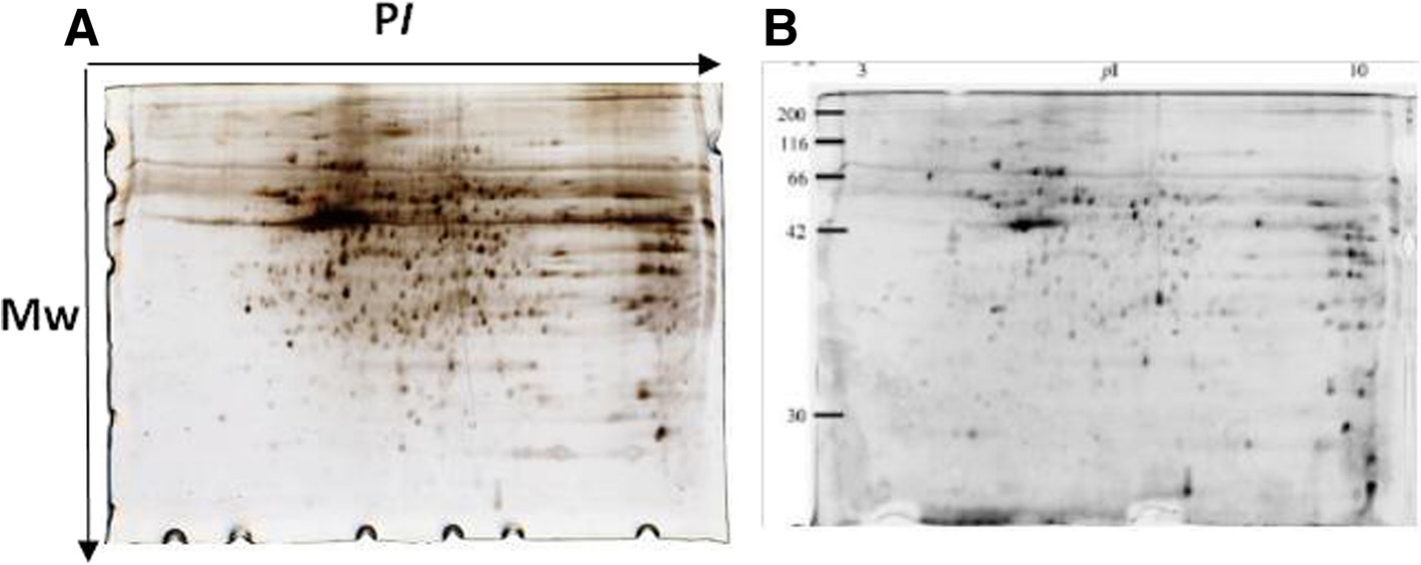
**Staining of 2-DE gels. A)** 24 cm two dimensional polyacrylamide gel electrophoresis of mouse colon protein stained by silver staining or **(B)** Deep purple flurophore dye. Visualization of B image was done using a Laser scanner [4].

Low dynamic range of proteins is one of the challenging problems encountered during 2-DE. Highly abundant peptides mask low abundant ones, which may be reflective of low abundant proteins. Moreover, the visualization of faint protein spots (low abundant) separated on 2-DE gels is also governed by staining sensitivity. For example, classic coomassie has a narrow dynamic range with detection limit of only about 100 ng. Colloidal coomassie is relatively higher in sensitivity with detection limit 10 ng. The dynamic range could be increased to a detection level below 1 ng with the availability of highly sensitive silver-staining method [12] and a diversity of fluorescence dyes such as SYPRO-Ruby and Deep purple flurophore dyes (Figure 2). Therefore, using sensitive stain increases protein sample dynamic range, leads to successful gel imaging, and finally leads to successful mass spectrometric identification and immunological validation [11]. Alternatively, depletion of highly abundant proteins such as albumin and hemoglobin significantly improves the dynamic range of 2-DE by allowing better focusing and mass spectrometric picking (see Prefractionation, enrichment, and depletion prior to 2-DE section). Therefore, the limited detection sensitivity of 2-DE does not cope with the actual dynamic range of protein concentration in cell and tissue extracts, or biological fluids [2]. Other solution to overcome the dynamic range problem is to load more protein sample and using a giant 2-DE gel (24 cm). However, this approach could result in production of overcrowded images with non well-separated spots.

#### Low throughput and labor- intensiveness

2-DE is labor-intensive and has a relative low throughput. The throughput of 2-DE is adequate for many small-scale basic research studies, but it may present a serious obstacle for projects that involve screening of a large number of clinical samples. Furthermore, 2-DE requires skills and experience to counteract any possible trouble shooting due to the procedural artifacts. Much care should be considered to minimize the variations during sample processing that lasts up to 3 days in larger gels.

### Recent advances and technologies associated with 2-DE

#### 2D- DIGE

The development of image technology has introduced differential imaging gel electrophoresis (DIGE) technique. This method was designed in an attempt to increase sensitivity and reproducibility of 2-DE using multiplexed fluorescent dyes- labeled protein samples. 2D-DIGE is based mainly on running more than one sample (maximum 3 samples) on a single gel at once to address the issue of gel-to gel variability. In this technique, different fluorescent cyanine (Cy) dyes are used for labeling proteins from different samples [25]. After mixing these samples in equal ratio and running them together as one sample, same protein from different samples migrates to the same position on the 2D gel where it could be easily explored and differentiated by the different fluorophore-labeled dye and imaged to calculate its abundance. 2D-DIGE is an important tool, especially for clinical laboratories involved in the determination of protein expression levels and disease biomarker discovery. When absolute biological variation between samples is the main objective, as in biomarker discovery, 2D-DIGE is one of the methods of choice.

#### Prefractionation, enrichment, and depletion prior to 2-DE

Protein sample prefractionation before 2-DE has been implemented to reduce sample complexity. As a result, the low abundance proteins present in these fractions will be clearly represented in 2-DE. Proteins identified in the prefractionated samples have a higher number of peptides. Moreover, low molecular weight proteins can be clearly detected when sample complexity is reduced [26]. Prefractionation ultimately increases loading capacity of samples onto the 2-DE gel and leads to better resolution, visualization and identification. Sample prefractionation can be performed in several different ways, broadly subdivided into three levels: cellular, subcellular and protein subfractionation [27]. For example, cellular extraction is a generalized approach to screen the whole proteome of a given sample. An extraction buffer containing urea and thiourea with NP-40 usually recovers most proteins [24]. In subcellular prefractionation, a density gradient (isopycnic) centrifugation method [28], or alternatively tissue strainers and buffers approach is used to homogenate a given tissue followed by using selective solvents to dissolve mitochondrial, ER-golgi, or nuclear proteins [29]. Protein subfractionation based on its physico-chemical properties is another level of fractionation. This method could be used to fractionate proteins based on their charge such as strong cation exchange (SCX) method [30] or based on their pI such as Liquid phase IEF Zoom® prefractionator [31]. Enrichment of protein of interest that exists in the sample in low abundance such as phosphorylated proteins (represents 3-4%) might be essential before 2-DE. For example, in one of our experiments to characterize tyrosine phosphorylated glomerular proteins related to slit diaphragm, samples were immunoprecipitated using protein A- Sepharose then subjected to 2-DE [32]. Depending on the nature of the sample, biological fluids such as plasma and urine may require removal of specific proteins (albumin or hemoglobin) to increase the resolution of separation before 2-DE. On the downstream mass spectrometric level, the excess of highly tryptic peptides generated from these abundant proteins bias the identification towards these proteins on the expense of lower abundant ones. In contrast, depletion increases peptides capture possibilities of low abundant proteins [33,34].

#### Blue native Gel electrophoresis for membrane proteins studies

Blue-Native polyacrylamide gel electrophoresis (Blue Native PAGE) was originally introduced by Schagger and von Jagow as a technique for separating enzymatically active membrane protein complexes under mild condition [35]. In this approach, the anionic dye Coomassie Brilliant Blue G-250 (5% w/v) is mixed with the protein sample prior to gel loading. This dye has the ability to provide negative charges to the surface of the protein. It induces a charge shift that improves solubilization of hydrophobic proteins especially membrane intrinsic electron/proton transfer complexes in mitochondria providing a global analysis of membrane proteomics. The dye binds to protein complexes and both migrate during electrophoresis. Finally, the gels are stained with coomassie dye again prior to mass analysis. A recent implementation for this technique was fruitful in identifying individual compounds within protein complexities of inflammasomes [36] and integrin and histone complexes in placenta [37]. In addition to its ability to unveil protein complexities, this methodology allows better separation of hydrophobic proteins (membrane proteins) as well [38].

#### 2-DE for post translational modifications (PTMs)

The analysis of protein post-translational modifications (PTMs) has become an important topic for the study of cell biology, disease treatment, and disease prevention. 2-DE provides a direct observation of protein PTMs in gels as well as its relative abundance. Publicly available web-based tools such as ProMoST [39] and JVirGel [40] could be used for that purpose. In addition, specific staining dyes for PTM monitoring were developed and became widely applied in the proteome studies. In phosphoproteome research, molecular Probes, Pro-Q® Diamond phosphoprotein gel stain is a breakthrough technology that provides a precise method for selectively staining phosphoproteins in polyacrylamide gels. Similarly, for glycoproteomics, commercially available fluorescent dye; Lissamine rhodamine B sulfonyl hydrazine (LRSH) was introduced to specifically stain the glycoproteins. This stain relays on periodate/Schiff base mechanism. Unlike conventional methods used for the characterization of PTMs such as the enrichment strategies either with antibodies or immobilized resin, lectin’s binding strategy, or enzymatic based techniques, 2-DE offers an analytical tool with high resolution and high reproducibility by taking the advantage of the well known change in pI and MW induced by many modifications [41].

## Outlines on the types and current availability of 2-DE

### First dimension electrophoresis

The first dimension electrophoresis can be performed using ionic substances, which reacts as acid or base and is termed as carrier ampholyte pH gradient. It could be blended and optimized for wide or more restricted pH ranges. Several techniques for IEF could be applied in the first dimension electrophoresis as explained below.

#### Conventional IEF

The conventional method of IEF depends on the carrier ampholyte where proteins migrate in a solution media until reaching the equilibrium state when its net charge equals to zero. Proteins that are in a pH region below its isoelectric point (pI) will be positively charged and will migrate towards the cathode. As it migrates through a gradient of increasing pH, however, the protein’s overall charge will decrease until the protein reaches the pH region that corresponds to its pI. At this point, the migration ceases. As a result, the proteins become focused into sharp stationary bands with each protein positioned at a point in the pH gradient corresponding to its pI. [2]. Although this conventional method is easy to prepare and do not require much casting equipments, it has a main disadvantage as the ampholytes have some susceptibility to flow towards cathode and this gradient flow usually causes a reduction in the reproducibility.

#### Immobilized pH gradient (IPG)

**Figure 3:**
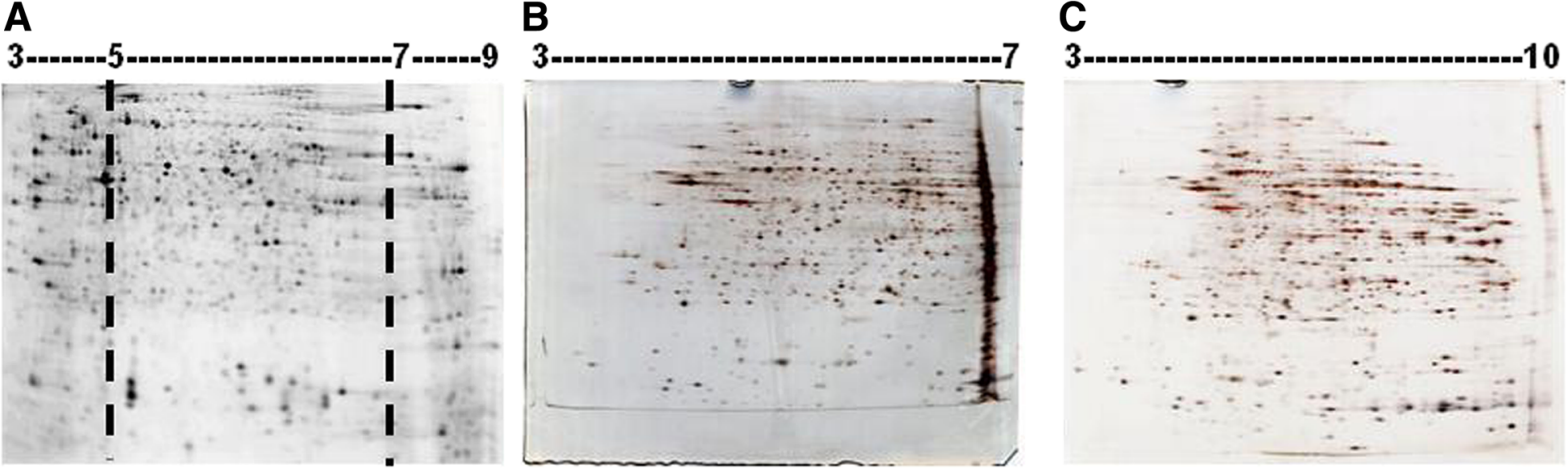
**Various IPG strips used in the first dimension step of 2- DE gels. (A)** Gel with non-liner (NL) IPG strips [pI 5–7]. **(B)** 8 cm gel with IPG strips [pI 3–7]. **(C)** Wide- ranged (pI 3–10) IPG strip.

Immobilized pH gradient strip (IPG) is an integrated part of polyacrylamide gel matrix fixed on a plastic strip. Co-polymerization of a set of non-amphoteric buffers with different chemical properties is included [2,42]. A ready- made IPG strips are available with different lengths and pI. Usually, short length IPG strips are used for fast screening while longer one for maximal and comprehensive analysis. Various models of 2- DE gels are shown in Figure 3. A commercial pre-casted acrylamide gel matrix co-polymerized with a pH gradient on a plastic strip results in a stable pH value over the traditional ampholyte method. It has an ability to avoid cationic accumulation and to produce a better-focused protein with less smearing [2,42]. There are many other advantages of using IPG strips over ampholytes such as reduced cathodic drift, higher mechanical strength as the strips are casted on a plastic backing that minimizing gel breakage, and higher protein loading capacity due to the sample loading method [17,43].

#### Non-equilibrium pH gel electrophoresis (NEPHGE)

Non-equilibrium pH gel electrophoresis (NEPHGE) technique was developed to resolve proteins with basic to extremely high pI (7.0 to 11.0) [1,44] that cannot be separated by the traditional method. In contrast, IPG method allows the protein molecules to move at different rate across the gel based on the charge and the volt hours setting that determine speed pattern and reproducibility [2]. A previous study comparing IPG and NEPHGE techniques showed that protein loss was higher in IPG-based method, especially for basic proteins. They found the reproducibility of spots was slightly better in NEPHGE-based method. About half of detected basic protein spots were not reproducible by IPG-based 2-DE, whereas NEPHGE-based method showed excellent reproducibility in the basic gel zone. The reproducibility of acidic proteins was similar in both methods [45].

### Second dimension SDS PAGE

This step separates proteins based on their molecular weight using a vertical electrophoretic device with either Laemmli buffer [46] or Tris- Tricine buffer [47]. Instead of loading protein sample within the wells, the first dimension-rehydrated strip is carefully placed on the top of the SDS-PAGE and sealed with agarose. Further technical details are described by Magdeldin *et al.*[4].

## The state of art in the analysis of 2-DE images

### 2- DE software analysis

**Figure 4:**
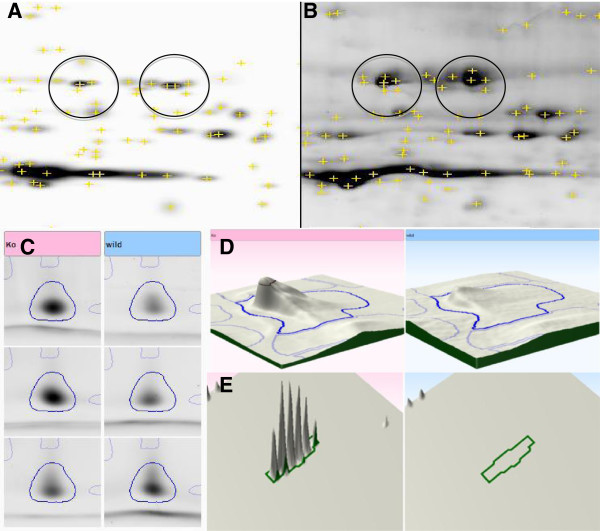
**Comparative analysis of gel image using PDQuest and Progenesis SameSpot.** Two different experimental groups were analyzed. As shown in **A** and **B**, a protein spot of F-cappig protein was recognized as a multiple spots by PDQuest when spot detection approach was used. **C** shows a precise detection of the same protein spot when images were aligned first (Progenesis SameSpot). The detected spot was confirmed later on a 2D **(D)** and 3D **(E)** views to be an actual fragmented peptide ions.

**Figure 5:**
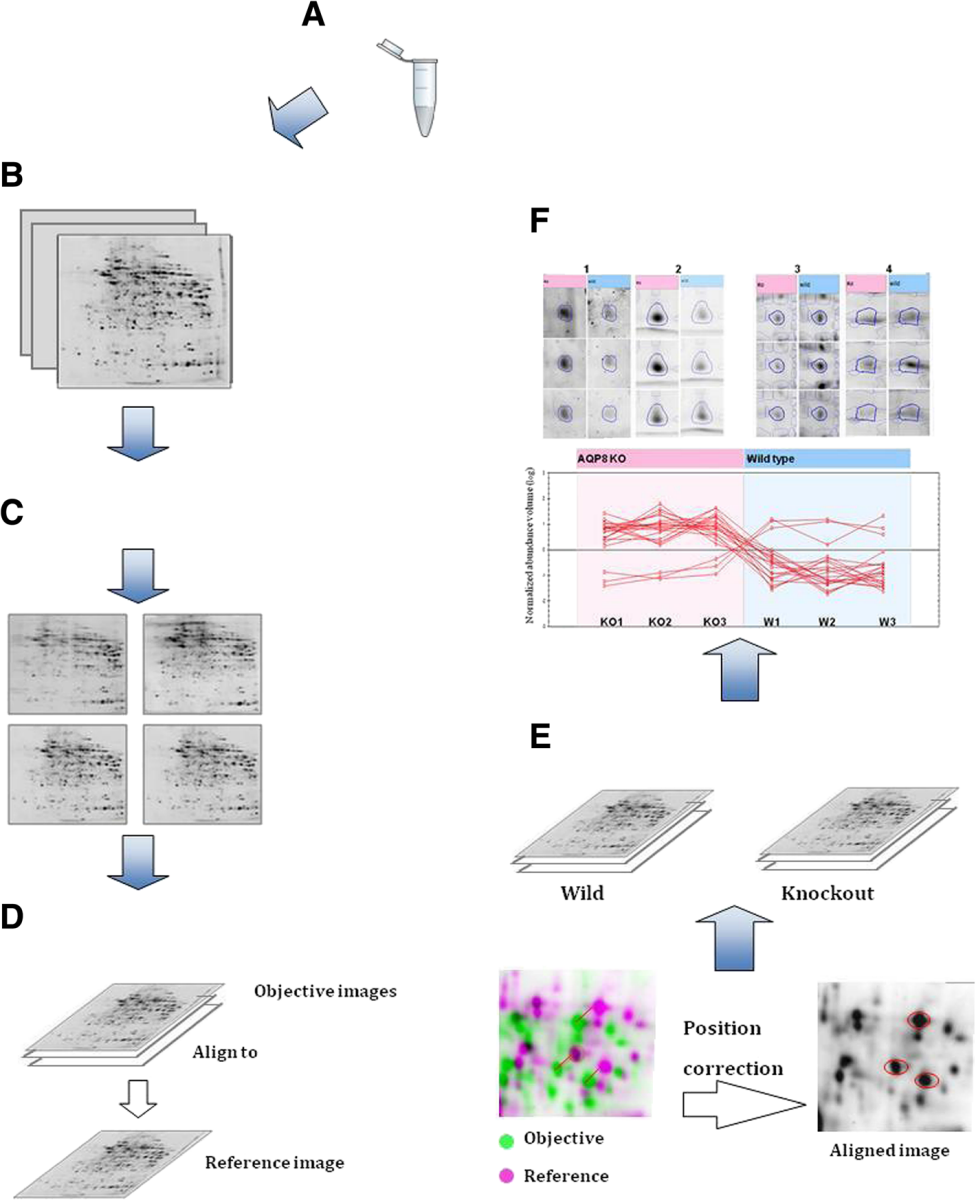
**Image analysis workflow of a 2-DE gel electrophoresis experiment using Progenesis SameSpots (Nonlinear Dynamics). A**. Sample preparation; **B**. 2-DE gel electrophoresis and gel staining; **C**. 2-DE image acquisition and image quality checked by the software automatically; **D**. Gel image alignment. A 2-DE gel image in the set of images for an experiment is manually set as the reference image (pink) by the user and then other 2-DE gel images (green) are aligned to the reference gel image one by one by a manual and/or automated way. **E**. After gel image alignment, the aligned images are grouped according to the experimental design. **F**. Extraction of proteins of interest. Spot volume normalization and calculation are performed by the software automatically.

Most of the software analysis workflow starts with either spot detection or image alignment. Examples of currently available software are listed in Table 1. In general, spot detection software (ex. PDQuest and Proteomweaver) sometimes lead to missing some data because of the mismatching error resulted due to shifted spots between gels or spots overlapping. Furthermore, false positive spots are commonly detected due to staining artifacts especially with poor technical skills. To avoid these problems, an alternative processing workflow approaches relay on aligning gel images before processing were developed (ex. Progenesis samespot and Decodon). This method increased the precision of spot detection, raised the accuracy of spot overlying, enabled alignment of several replicates, and saved time. In a practical example illustrated in Figure 4, same gel images of 2 groups were analyzed using PDquest and progenesis samespot software that utilize initial step of spot detection or image alignment, respectively. The result showed inaccuracy of spot detection in the analysis starting with spot detection by detecting many false positive spots. On contrary, precise spots were recognized when gel images where aligned first. This improvement in gel image processing reduced variability between gels and increased the power to detect differentially expressed spots with less effort of manual editing. The key steps of the workflow of most of these automated methods are as following; image quality control, image alignment, spot detection, automatic analysis, editing of spot detection, review the results, statistical analysis, calibration of spots against either a molecular weight ladder or known proteins, spot picking, and importing the protein ID (Figure 5). These automated methods are helpful for gel identification particularly in the quantitative proteomics. They primarily analyze biomarkers by quantifying individual proteins and showing the separation between one or more protein spots on a scanned image of a 2-DE gel. Additionally, they match spots between gels of similar samples. For example, proteomic differences between early and advanced stages of an illness. However, any change or failure in the spot identification may affect the peptide identification in the downstream proteomics as explained later in the challenges section (Challenges in the analysis of the 2-DE images).

**Table 1:** Current available commercial software used for 2-DE gel image analysis

**Company**	**Software name**	**Image analysis approach**
**Bio-Rad**	PDQuest	Spot detection first
	Proteomweaver	Spot detection first
**GE Healthcare**	Decyder 2D	Spot detection first
**GE Healthcare**	ImageMaster 2D platinum	Spot detection first
**GeneBio**	Melanie	Spot detection first
**Applied Maths NV**	BioNumerics	Spot detection first
**Ludesi**	Redfin	Spot detection first
**Ludesi**	Gel IQ*	Spot detection first
**Compugen**	Z3 (discontinued)	Image alignment first
**Decodon**	Delta2D	Image alignment first
**Nonlinear dynamics**	Progenesis SameSpots	Image alignment first
**Sourceforge**	Flicker*	Image alignment first

*Open source or free of charge.

### 2-DE proteome databases

2-DE proteome database websites allow experimental data to be uploaded and disseminated for public. They also help to generate reference maps for normal and diseased cases. These databases display annotated and representative reference map for a variety of cell and tissue types. A list of publicly available databases is found at http://www.expasy.org/ch2d-index.html. The Swiss- 2D PAGE website highlights the World- 2D PAGE database server, the World-2 D PAGE Portal, and the World- 2D PAGE repository, which displays the publication links of each reference map and the experimental condition [48]. So far, it is one of the largest databases for 2-DE. It contains more than 36 different 2-DE reference proteome maps from 7 different species including *S. Cerevisiae* and *E. coli* as well as many different human tissues and organs such as kidney and liver [48]. The database annotates protein identification, protein patho-physiological function, physicochemical properties (MW and pl, amino acid composition and peptide masses), and citations. Recently, many other 2-DE reference maps are available online e.g. (http://web.mpiib-berlin.mpg.de/cgi-bin/pdbs/2d-page/extern/index.cgi and http://www.gelbank.anl.gov).

### Challenges in the analysis of the 2-DE images

Although bioinformatics software made 2-DE gel analysis much easier, several challenges remain in order to achieve a non-biased, accurate, and reproducible result.

#### Before software analysis

The key role in a successful computer- based gel imaging relays on the quality of the raw image itself. Therefore, it is necessary to use a high resolution scanner. CCD cameras are time consuming but could be used for several stained gels. laser scanners are more accurate and generate high resolution raw images. In general, image resolution of 100–150 in TIEF format is enough for quantitative analysis. In comparative analysis, it is essential to reproduce replicate gels with minimal noise and background for correct quantification. Certain filters might be applied with care for gel optimization prior to analysis.

#### During the process of analysis

Differences in the spot positions between gels are major challenging issue in image processing because they impede accurate spot matching. The key step for overcoming spot shifting is to perform a gel image alignment in which certain landmark spots are first pinned between all gels. In a next step, the software tries to align other spots based on these landmarks. However, in some cases, complete alignment could not be obtained if the two patterns are so different. Therefore, in such case, one should avoid excessive manual interventions because this would worsen the reliability of the image control and the reproducibility of the operation between different users. Because of the quantification and the normalization of the spot intensity, one should realize that the relationship between the original protein quantity in the sample and the measured spot intensity is affected by various intervening factors. For example, sample loss occurring during the IEF or while transferring to the second dimension. Given the biochemical diversity of the protein molecules, it is expected that there are some proteins with a nonlinear relation between concentration and intensity. Therefore, one should expect to obtain relatively quantitative results referring to same protein species coming from different samples.

## Concluding remarks

Protein separation is a core part of proteomics analysis. 2-DE is a basic and fundamental procedure to fractionate and visualize protein complexes. The 2-DE method is superior to visualize each protein as a spot that can be interpreted by its abundance, location, or even its presence or absence. This visual- based result is in most cases confident. Meanwhile, 2-DE procedures need experience and optimization of skills. With the ongoing development and modifications applied to this sophisticated technique, 2-DE is expected to be less labor, more informative, sensitive, rapid, and easily applied.

## Competing interest

The authors declare that they have no competing interest.

## Authors’ contributions

SM, SE, YY, BX, YZ, ZZ, IL, EY drafted the manuscript. TY edited and approved the manuscript. All authors read and approved the final manuscript.
